# AGEs-Induced IL-6 Synthesis Precedes RAGE Up-Regulation in HEK 293 Cells: An Alternative Inflammatory Mechanism?

**DOI:** 10.3390/ijms160920100

**Published:** 2015-08-25

**Authors:** Andreea Iren Serban, Loredana Stanca, Ovidiu Ionut Geicu, Anca Dinischiotu

**Affiliations:** 1 Department of Preclinical Sciences, Faculty of Veterinary Medicine, University of Agronomical Sciences and Veterinary Medicine Bucharest, 105 Splaiul Independentei, district 5, Bucharest 050097, Romania; E-Mails: lory_stanca@yahoo.com (L.S.); yo3hfh@yahoo.com (O.I.G.); 2 Department of Biochemistry and Molecular Biology, Faculty of Biology, University of Bucharest, 91–95 Splaiul Independentei, District 5, Bucharest 050095, Romania; E-Mail: ancadinischiotu@yahoo.com

**Keywords:** advanced glycation end products, heat-shock proteins, HEK 293 cells, inflammation, oxidative stress

## Abstract

Advanced glycation end products (AGEs) can activate the inflammatory pathways involved in diabetic nephropathy. Understanding these molecular pathways could contribute to therapeutic strategies for diabetes complications. We evaluated the modulation of inflammatory and oxidative markers, as well as the protective mechanisms employed by human embryonic kidney cells (HEK 293) upon exposure to 200 μg/mL bovine serum albumine (BSA) or AGEs–BSA for 12, 24 and 48 h. The mRNA and protein expression levels of AGEs receptor (RAGE) and heat shock proteins (HSPs) 27, 60 and 70, the activity of antioxidant enzymes and the expression levels of eight cytokines were analysed. Cell damage via oxidative mechanisms was evaluated by glutathione and malondialdehyde levels. The data revealed two different time scale responses. First, the up-regulation of interleukin-6 (IL-6), HSP 27 and high catalase activity were detected as early as 12 h after exposure to AGEs–BSA, while the second response, after 24 h, consisted of NF-κB p65, RAGE, HSP 70 and inflammatory cytokine up-regulation, glutathione depletion, malondialdehyde increase and the activation of antioxidant enzymes. IL-6 might be important in the early ignition of inflammatory responses, while the cellular redox imbalance, RAGE activation and NF-κB p65 increased expression further enhance inflammatory signals in HEK 293 cells.

## 1. Introduction

Advanced glycation end products (AGEs) are the result of non-enzymatic glycation processes, a cascade of events that begins with the reversible formation of a Schiff base that undergoes rearrangement to an Amadori product through a reaction between reducing sugars and amino groups of proteins [1]. This compound is slowly degraded to a plethora of compounds of various chemical structures including: *N*^ε^-carboxymethyllysine (CML) [2], pentosidine [3], and glucosepan [4], some of which possess distinct fluorescence and a propensity to crosslink proteins [1,5]. In diabetes, and natural aging, AGEs accumulate in different target tissues, playing a role in the pathogenesis of various complications, particularly in nephropathy through pathways dependent or independent of AGEs receptors [6,7,8,9].

Diabetes complications are essentially linked with chronic inflammatory response and significant disturbance of the redox equilibrium, resulting in oxidative stress. These two aspects have been linked by pattern recognition receptors (PRR), which can account for both the inflammatory component and the oxidative stress [10]. Research advances in the field of AGEs receptor (RAGE) ligands have shown that AGEs are not the sole activator of RAGE, as this pattern recognition receptor is able to bind numerous damage-associated molecular patterns (DAMPs) molecules [11,12,13]. Ligand recognition by RAGE does not result in ligand uptake and degradation [7], instead, it triggers the activation of RAGE pathways including Jak/Stat, NF-κB, and mitogen activated protein kinase (MAPK), such as p38, extracellular regulated (ERK)-1/2 and c-Jun N-terminal kinase (JNK) [9].

The plethora of RAGE ligands involves it in either physiological contexts [14] or pathological ones [7,8]. Chronic RAGE activation (often associated with a hyperglycaemic context) is involved in the onset and upkeep of the inflammatory response [7] and generation of a pro-fibrotic state [15]. Moreover, AGEs–RAGE interaction was also shown to promote increased ROS generation [16] and subsequent activation of MAPK/p42/44 and NF-κB, resulting in an enhanced expression of RAGE in a positive autoregulatory loop [6].

In this ROS-dependent mechanism of AGEs-induced nephropathies, p66Shc was shown to be essential, being involved in the activation of NADPH oxidase and NF-κB [17]. Therefore, the *in vitro* and *in vivo* studies provide firm evidence that RAGE activation through its ligand is central to the onset and progression of diabetes complications. However, the limited efficacy of therapeutical interventions based on RAGE blocking or AGEs inhibition [14] seem to overlook other mechanisms involved in AGEs-activated routes. The onset and development of diabetic nephropathies is dependent on both its oxidative and inflammatory components, and the study of the molecular links merits further attention, hence our major motivation to conduct this work. A promising direction could be the function of heat shock proteins (HSPs), which exhibit anti-oxidative [18] and both pro- [19] and anti-inflammatory [20] properties, depending on the biochemical context.

The human embryonic kidney (HEK) 293 cell line was demonstrated to maintain important renal-specific properties [21,22]. HEK 293 have epithelial morphology, with an apical *zonae occludentes*, and less pronounced brush-border [21]. Also, they display several features of renal distal tubular cells, such as sensitivity to vasoactive intestinal peptide, osmotic stress and isoproterenol and the expression of renal natriuretic peptide. They are also insensitive to Arg8–vasopressin and present low levels of alkaline phosphatase activity [22]. All these characteristics indicate HEK 293 cells could be a good renal cellular model system for deciphering the mechanisms that underlie diabetic nephropathy.

We set out to evaluate the effects induced by AGEs–BSA (bovine serum albumin) on HEK 293 cells, regarding the modulation of inflammatory and oxidative markers as well as the protective mechanisms employed, including heat shock proteins and antioxidant enzymes.

## 2. Results and Discussion

### 2.1. The Degree of BSA Glycation

The glycated and unglycated BSA solutions used here were previously characterized by our group using fast protein liquid chromatography (FPLC), SDS-PAGE and fluorescence spectroscopy. Briefly, we showed by FPLC analysis that the glycated BSA monomer’s molecular weight increased by 5.11 kDa, and also dimers were observed in glycated BSA. SDS-PAGE gels indicated an 8 kDa increase in the molecular weight of the glycated BSA monomers. Glycated BSA fluorescence significantly increased, indicating both the presence of pentosidine-like compounds and Maillard-like compounds [15]. Additionally, we have now quantified the AGEs content, and report that glycated BSA contained 4.8 ng AGEs/μg total protein, while in unglycated BSA we detected 1.5 ng AGEs adducts/μg total protein.

### 2.2. Cell Viability

AGEs–BSA exposure did not induce significant changes in HEK 293 cell viability. Cell viability was 92.2% ± 3.3%, 94.6% ± 2.7% and 93% ± 2.3% in the case of AGEs–BSA exposed cells for 12, 24 and 48 h respectively, while at the same time points, control cells viability was 93.7% ± 1.5%, 95% ± 4.5% and 96% ± 3.5%.

### 2.3. Inflammatory Response Evoked in HEK 293 Cells

The first changes observed in HEK 293 cells exposed to AGEs–BSA were associated with the immune response. An interesting expression profile was obtained for IL-6, the only cytokine that was elevated (by 3.31-fold) after 12 h in AGEs–BSA exposed cells and returned to control levels thereafter. In the conditioned media from AGEs–BSA exposed cells, the level of IL-6 increased by 10.37, 3 and 2.5-fold after 12, 24 and 48 h respectively. IL-8 was detected in cell lysate and conditioned media, and its levels increased in both cases in a time dependent manner, and reached a maximum of 1.94 and 2.78-fold respectively after 48 h of AGEs–BSA exposure (Table 1).

**Table 1:** Cytokine levels in HEK 293 lysate and in culture medium after exposure to AGEs or unglycated BSA.

Cytokine pg/mL	Treatment		Cell Lysate			Cell Culture Medium		
			12 h	24 h	48 h	12 h	24 h	48 h
IL-2	200 μg/mL	BSA	n.d.	n.d.	0.54 ± 0.05	n.d.	n.d.	n.d.
		AGEs	n.d.	n.d.	1.27 ± 0.15	n.d.	n.d.	n.d.
	Fold change		−	−	2.34 **	−	−	−
IL-4	200 μg/mL	BSA	n.d.	n.d.	0.3 ± 0.08	n.d.	n.d.	n.d.
		AGEs	n.d.	n.d.	0.74 ± 0.06	n.d.	n.d.	n.d.
	Fold change		−	−	2.48 ***	−	−	−
IL-6	200 μg/mL	BSA	0.72 ± 0.12	0.42 ± 0.12	0.26 ± 0.08	7.67 ± 0.53	5.45 ± 0.22	4.65 ± 0.13
		AGEs	2.37 ± 0.29	0.37 ± 0.13	0.23 ± 0.01	79.49 ± 5.01	16.41 ± 2.3	11.61 ± 1.54
	Fold change		3.31 ***	0.89	0.88	10.37 ***	3.01 ***	2.5 **
IL-8	200 μg/mL	BSA	10.13 ± 2.4	11.47 ± 1.44	33.9 ± 2.34	54.4 ± 4.21	75.82 ± 6.16	85.82 ± 7.2
		AGEs	11.29 ± 3.76	20.02 ± 3.45	65.69 ±5.98	74.4 ± 4.03	111.69 ± 9.21	237.6 ± 12.4
	Fold change		1.11	1.75 ***	1.94 ***	1.37 *	1.47 *	2.78 ***
IL-10	200 μg/mL	BSA	n.d.	n.d.	0.92 ± 0.13	n.d.	n.d.	n.d.
		AGEs	n.d.	n.d.	2.43 ± 0.17	n.d.	n.d.	n.d.
	Fold change		−	−	2.64 ***	−	−	−
GM-CSF	200 μg/mL	BSA	7.69 ± 1.49	8.34 ± 0.75	9.61 ± 0.24	n.d.	n.d.	n.d.
		AGEs	9.72 ± 0.59	11.73 ± 0.75	18.33 ± 0.88	n.d.	n.d.	n.d.
	Fold change		1.26 *	1.41 ***	1.91 ***	−	−	−
IFN-γ	200 μg/mL	BSA	14.68 ± 1.52	12.24 ± 0.98	12.6 ± 0.28	n.d.	n.d.	n.d.
		AGEs	17.87 ± 1.11	22.08 ± 0.02	41.81 ± 3.5	n.d.	n.d.	n.d.
	Fold change		1.22 **	1.8 ***	3.32 ***	−	−	−
TNF-α	200 μg/mL	BSA	n.d.	n.d.	0.86 ± 0.03	n.d.	n.d.	n.d.
		AGEs	n.d.	n.d.	2.84 ± 0.05	n.d.	n.d.	n.d.
	Fold change		−	−	3.17 ***	−	−	−

n.d.—not detected; * p < 0.05; ** p < 0.01; *** p < 0.001.

**Figure 1:**
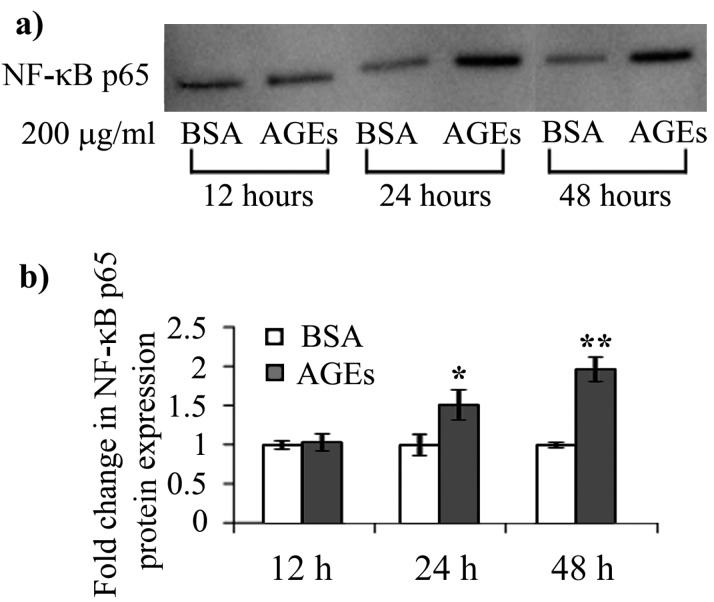
NF-κB p65 protein expression levels (**a**) Representative immunoblot membrane; (**b**) Combined densitometry data of three individual experiments, indicating fold changes in the protein expression of NF-κB p65, normalized to the total proteins transferred onto the membrane. BSA: bovine serum albumin; AGEs: advanced glycation end products; * *p* < 0.05; ** *p* < 0.01.

The expression of IL-6 cytokine was recently found to be associated with p38-MAPK signaling rather than NF-κB [23]. Our results strengthen this idea, as IL-6 level in cell lysates and in conditioned media registered the maximum increases after 12 h of exposure to AGEs–BSA, indicating that the mechanisms involved in IL-6 expression were active at the 12 h exposure interval, a time point when we found no significant NF-κB p65 up-regulation (Figure 1). Secreted IL-6 elicits an inflammatory response, induced through IL-6 receptor (IL-6R), found in inflammatory cells and also expressed by HEK 293 cells [24]. IL-6 recognition by IL-6R was shown to induce pro-inflammatory STAT3 activation both *in vivo* and *in vitro*, in various cell types, including HEK 293 cells [25,26,27]. Additionally, in HEK 293 cells, STAT3 activation by IL-6 was shown to induce IL-8 expression [26], a mechanism which supports our results, and reveals an early IL-6 up-regulation preceded IL-8 expression after 12 h of AGEs–BSA exposure (Table 1). The return of IL-6 to control levels after 48 h of AGEs–BSA exposure (Table 1), might indicate that cellular signaling in HEK 293 might also have an anti-inflammatory component. Although the absolute concentrations were low, IL-2, IL-4 and IL-10 detected in cell lysates at the 48 h time interval were significantly higher than controls (Table 1), and could additionally contribute to dampen the inflammatory signals [28,29].

Granulocyte-macrophage colony-stimulating factor (GM-CSF) and interferon gamma (IFN-γ) were detected only in cell lysates, and their expression increased in a time dependent manner, by 1.26-, 1.41- and 1.91-fold after 12, 24 and 48 h respectively in the case of GM-CSF and by 1.22-, 1.8- and 3.32-fold in the case of IFN-γ. GM-CSF expression was shown to increase during cellular stress, and elicit an anti-apoptotic function, conferring tolerance to oxidative stress by activating HSF-1 via STAT3 [30]. Furthermore, it was demonstrated that GM-CSF is involved in maintaining mitochondrial integrity, via Akt activation [31]. Experimental evidence suggested that TGF-β1 also participates in the activation of the phosphoinositide-3-kinase (PI3K)/Akt pathway [32] and we have previously demonstrated that TGF-β1 expression is quickly and strongly induced in HEK 293 cells after exposure to AGEs–BSA [15].

Interferon synthesis is generally considered a characteristic of the myeloid lineage cells, although other cells, like pulmonary epithelial cells, were shown to have this capacity [33]. Epithelial pulmonary cells also possess high basal RAGE expression levels [34], which could link RAGE signaling and interferon expression. In our case, IFN-γ expression, induced by AGEs–BSA exposure in a time dependent manner, could possess a biological activity similar to that of extracellular IFN-γ [35] and could act to temper the immune response by inducing STAT1 activation and STAT3 deactivation, as described in other reports [36].

### 2.4. AGEs–BSA Exposure Activates NF-κB and RAGE Expression

NF-κB activation via increased expression of p65 protein was associated with chronic RAGE stimulation by AGEs and is characteristic of diabetes complications [37]. In HEK 293 cells exposed to 200 μg/mL AGEs–BSA we registered significant increases in p65 protein expression, by 1.5- and 1.95-fold after the 24 and 48 h exposure intervals (Figure 1).

**Figure 2:**
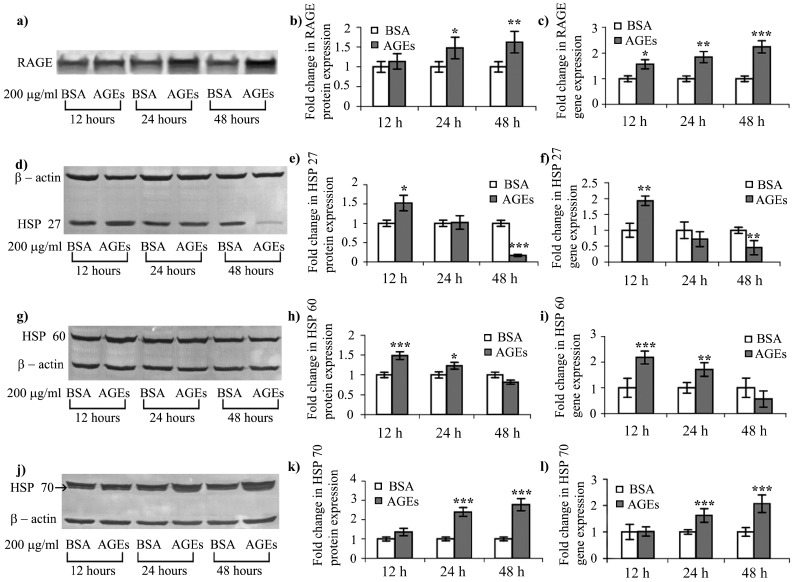
Gene and protein expression of AGEs receptor (RAGE) and heat shock proteins modulated by AGEs (**a**,**d**,**g**,**j**) Representative blotted membranes indicating RAGE, HSP 27, HSP 60 and HSP 70 protein expression; (**b**,**e**,**h**,**k**) Combined densitometry data of three blotted membranes indicating fold changes in protein expression of RAGE (reported to total proteins loaded), HSP 27, HSP 60 and HSP 70 (reported to β-actin); (**c**,**f**,**i**,**l**)-relative expression ratio of *RAGE*, *HSP 27*, *HSP 60* and *HSP 70* target genes (*GAPDH*, housekeeping gene). HSP: heat shock protein; * *p* < 0.05; ** *p* < 0.01; *** *p* < 0.001.

RAGE levels were up-regulated in 200 μg/mL AGEs–BSA milieu, in a time dependent manner. Protein expression increased significantly by 1.3- and 1.6-fold after 24 and 48 h respectively (Figure 2a,b), while the mRNA expression level increased by 1.41-, 1.67- and over 2-fold after 12, 24 and 48 h respectively (Figure 2c). RAGE up-regulation is known to result in perpetuated NF-κB signaling activation [6], which is also involved in regulating cytokine expression. In our case, AGEs–RAGE induced NF-κB p65 expression could additionally contribute to the increase of IL-8 cytokine expression levels, complementing the effects of IL-6 discussed in Section 2.3 (Figure 1 and Figure 2a–c and Table 1).

**Figure 3:**
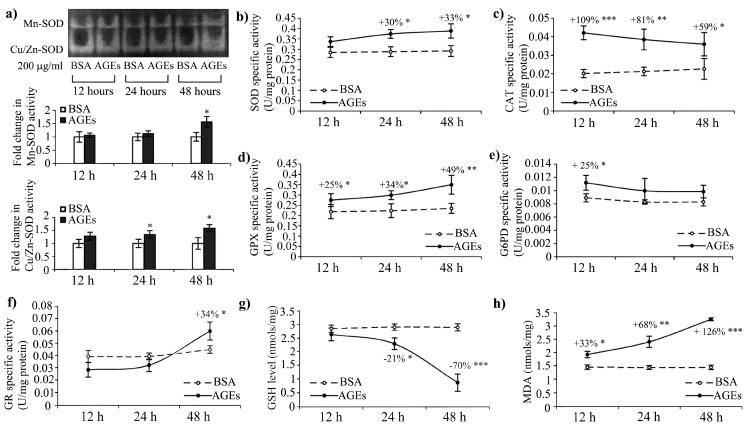
Antioxidative defence in HEK 293 cells and oxidative stress induced by AGEs exposure. (**a**) Representative zymogram indicating the activity of SOD enzymes and combined densitometry data of three zymograms indicating fold changes in Mn–SOD and Cu/Zn–SOD activities. The specific activities of total (**b**) SOD; (**c**) CAT; (**d**) GPX; (**e**) G6PD; (**f**) glutathione reductase (GR) antioxidative enzymes; (**g**) GSH and (**h**) advanced lipid oxidation end products (MDA, malondialdehyde). In (**b**–**h**), when statistically significant, the percent change values were given. * *p* < 0.05; ** *p* < 0.01; *** *p* < 0.001.

**Figure 4:**
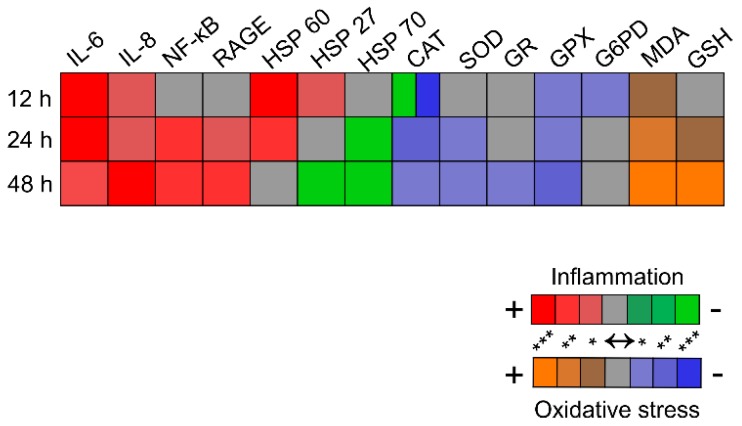
Heat plot indicating the parameters changes and their pro-/anti-inflammatory or the pro-/anti-oxidative contributions, as discussed in the text. The amplitude of the changes is color-coded based on the *p* value of statistic significance. The relative changes in the extracellular level of cytokines, protein expression levels of NF-κB, RAGE and HSPs and specific enzymatic activity are shown. * *p* < 0.05; ** *p* < 0.01; and *** *p* < 0.001.

RAGE’s activation by its ligands is known to mediate ROS generation via cytosolic pathways by NADPH oxidase or mitochondrial electron transport chain [17,38]. ROS formation further activates NF-κB, a scenario that in our case is favoured under diminished catalase activity and GSH depletion, which we report starting with the 24 h exposure (Figure 3 and Figure 4).

### 2.5. HSPs Response to AGEs–BSA Exposure in HEK 293 Cells

The HEK 293 cells responded to 200 μg/mL AGEs–BSA with an interesting HSPs interplay. The protein expression of HSP 27 was significantly increased by 1.53-fold after 12 h, and began to decrease thereafter, reaching a level almost six-fold lower compared to the control level after 48 h of AGEs–BSA exposure (Figure 2d,e). This profile was confirmed by real time PCR data (Figure 2f). In AGEs–BSA exposed cells, HSP 60 showed a pattern similar to HSP 27, its protein and gene expression being elevated after 12 h by 1.49- and 2.18-fold respectively compared to BSA exposed cells. Both protein and gene expression levels returned to control values after 24 h (Figure 2g–i). The increased expression of both HSP 27 and HSP 60 after 12 h of AGEs–BSA exposure could be induced by STAT3 activation, which was shown to stabilize HSF-1 [30], a chaperone transcription factor, thus encouraging heat shock protein synthesis. The raised expression level of HSP 60 after 12 h of AGEs–BSA exposure (Figure 2g–i), might contribute to NF-κB signalling activation, as previous research has suggested [39]. Moreover, HSP 27 is known to be a component of the MAPK signaling pathway, and stabilizes IL-6 mRNA, supporting its protein expression [19]. HSP 27 expression profile is particularly interesting, as our results have shown an inhibition after 48 h of AGEs–BSA exposure (Figure 2d–f). Decreased expression of HSP 27 was previously associated with p38-MAPK inhibition [40] and elicits an anti-inflammatory response [41].

HSP 70 protein and gene expression profiles increased in a time-dependent manner, starting with the 24 h AGEs–BSA exposure interval, by 2.4- and 1.62-fold respectively, while after 48 h both protein and gene expression levels increased by about 2-fold (Figure 2j–l). Intracellular HSP 70 expression has been demonstrated to have a net pro-survival effect, exerting cytoprotective functions, by inhibiting apoptosis at multiple points and also by inhibiting NF-κB inflammatory pathways [42].

### 2.6. Oxidative Stress Induced by AGEs–BSA Exposure and the Antioxidant Response of HEK 293 Cells

Proximal signaling events after AGEs recognition by RAGE, include an induction of ROS, that stimulates inflammation through various signaling pathways [43]. The cytosolic and mitochondrial superoxide dismutase (SOD) enzymes analyzed by zymography contributed to the total SOD activity at different time points. Cu/Zn–SOD activity was more accentuated after 12 h, while Mn–SOD activity increased after the 48 h AGEs–BSA exposure interval (Figure 3a). The total SOD activity was increased beginning with the 12 h interval by 18%, and remained elevated by 33% after 48 h (Figure 3b). Catalase (CAT) response was prompt; its activity increased by over 2-fold after the 12 h exposure, and continued to be elevated even after 48 h (Figure 3c).

AGEs stimulation was shown to induce Akt activation in HEK 293 cells [44], probably with the contribution of GM-CSF increased levels (Table 1). Akt is known to inhibit apoptosis [45], to activate NF-κB [46,47] and antioxidant enzymes such as Mn–SOD [48]. Our results showed a significant increase of Mn–SOD activity after 48 h of exposure to AGEs–BSA (Figure 3a). Also, there seems to be an inverse relation between Akt activation and catalase expression [49,50], thus the high catalase activity (after 12 h its activity increased over 2-fold compared to control cells) might have an inhibitory effect on NF-κB signaling, a pathway co-stimulated by H_2_O_2_ [51]. Catalase activity is known to increase in the presence of its substrate [52], and the strongly up-regulated activity we have noted, especially after 12 h of AGEs–BSA exposure, could explain both H_2_O_2_ generation in HEK 293 cells and the delayed NF-κB activation (Figure 1 and Figure 3c), favourable under diminished catalase activity [48,51]. The cytokine tumour necrosis factor α (TNF-α) mediates the downregulation of catalase expression [51]. This is in agreement with our data, which indicated increased levels by 3.17-fold after 48 h of AGEs–BSA exposure (Table 1), a time interval that is associated with the lowest values of CAT activity we have registered (Figure 3c).

The activity of glutathione peroxidase (GPX), another ROS scavenging enzyme that can also detoxify H_2_O_2_, had an opposite profile compared to that of CAT (Figure 3c,d). Its activity profile increased gradually during the intervals analyzed, and reached the highest increase, 50% over the control level, after 48 h (Figure 3d). Interestingly, GPX is known to be induced by NF-κB [48], and this might explain why GPX and SOD activities were increased after 48 h of AGEs–BSA exposure, when we observed the strongest NF-κB p65 activation (Figure 1 and Figure 4).

Glucose-6-phosphate dehydrogenase (G6PD) is the main producer of NADPH, an important intracellular reductant. G6PD activity was significantly increased by 25% after 12 h of exposure to AGEs–BSA, while the longer exposure intervals revealed that the activity returned to control levels (Figure 3e). In contrast, glutathione reductase (GR) activity increased only after 48 h of AGEs–BSA exposure, by 35% (Figure 3f).

AGEs were previously shown to inhibit G6PD activity [53] and, in our case, the 24 and 48 h exposure intervals reduced G6PD activity to levels similar to control cells (Figure 3e), which most probably cannot confer proper cellular antioxidative protection [54].

The cellular antioxidative resources were drained, as the reduced glutathione (GSH) gradually diminished, being diminished by 70% after 48 h (Figure 3g). Additionally, malondialdehyde (MDA) levels increased by 75% and 129% at the 24 and 48 h intervals (Figure 3h), suggesting advanced lipid peroxidation occurred in AGEs–BSA exposed HEK 293 cells.

Literature data have demonstrated that expression levels of several cytokines are closely bound with the reduced glutathione depletion and that thiols can inhibit the activation of different transcription factors (NF-κB, AP-1 and/or NF-IL-6) depending on the activator used [55]. In our case only 70% GSH depletion was reached after the 48 h AGEs exposure, which could imply that at a later time point, TNF-α expression might increase. The above-referenced work also demonstrated that although total depletion of glutathione significantly increases TNF-α and IL-8 synthesis, it does not modulate IL-6 secretion. This agrees very well with our data, which showed that the only cytokine strongly expressed early in the experiment timeline is IL-6 (Table 1 and Figure 4).

Stimulated by the early (12 h) IL-6 expression and the prolonged (24 h) AGEs exposure, NF-κB p65 expression is activated and IL-8 and RAGE protein expression significantly increase. The late and moderate activation (48 h) of SOD, GR and GPX on the one side and the decrease of CAT and G6PD from initially high levels (12 h) on the other side contribute to oxidative stress, as shown by GSH depletion and MDA rise. The decrease of HSP 27 and 60 expression levels and the increase of HSP 70 may contribute to negative regulatory feed-back mechanisms to inhibit inflammation, although the installment of oxidative stress has a pro-inflammatory effect (Figure 4).

## 3. Experimental Section

### 3.1. AGEs–BSA Preparation and the Assessment of BSA Glycation

Glycated and unglycated BSA were obtained and characterised by gel-filtration chromatography, SDS-PAGE and fluorescence spectroscopy as previously described [15]. Additionally, the content of N(6)-carboxymethyllysine (CML), pentosidine and other AGEs compounds in glycated BSA or unglycated BSA (control) were evaluated with the Advanced Glycation End Product ELISA kit (Cell Biolabs, San Diego, CA, USA) according to the manufacturer’s instructions. The AGEs content of our samples were determined using a standard curve with AGEs–BSA control provided in the kit. Both AGE–BSA and BSA used in cell treatments were endotoxin-free (E-toxate kit, Sigma-Aldrich, St. Louis, MO, USA).

### 3.2. Cell Culture and Treatment

HEK 293 (ATCC, Manassas, VA, USA) cells were grown in DMEM supplemented with 1× antibiotic-antimycotic solution and 10% fetal bovine serum (Life Technologies, Carlsbad, CA, USA), in 5% CO_2_ atmosphere. The cells were gradually acclimatized to serum free conditions. HEK 293 were exposed for 12, 24 and 48 h to 200 μg/mL AGEs–BSA or BSA (control).

After treatment with 200 μg/mL AGEs–BSA or BSA for 12, 24 and 48 h, cellular viability was determined using the TC20 automated cell counter (Bio-Rad Laboratories, Hercules, CA, USA).

### 3.3. Preparation of Conditioned Media and Quantitation of Inflammatory Cytokines

At the end of the incubation periods, the cell culture supernatants were harvested and centrifuged to remove cell debris. The conditioned media was concentrated using the 3 kDa cut off membrane (Millipore, St. Charles, MO, USA), and samples were aliquoted and immediately frozen at −80 °C. The concentrated conditioned media were used for cytokine expression assay.

The extracellular and intracellular levels of IL-2, IL-4, IL-6, IL-8, IL-10, GM-CSF, IFN-γ and TNF-α cytokines were evaluated using the Bio-Plex Pro Human Cytokine 8-plex panel (Bio-Rad Laboratories, Hercules, CA, USA). Cell lysates and the conditioned media samples had a total protein concentration of 600 μg/mL. The cytokine expression level was analyzed using the Bio-Plex MAGPIX System (Bio-Rad Laboratories), and concentrations of each cytokine were determined using Bio-Plex Manager software version 6.0 (Bio-Rad Laboratories).

### 3.4. Antioxidant Enzymes Activity

SOD activity was assessed using the method described by Paoletti and Mocali [56]. One unit (U) of SOD activity was defined as the amount of enzyme that inhibited NADPH oxidation by 50% compared to oxidation rate in the reagent blank.

CAT activity was assessed following Aebi’s method [57]. One unit of CAT activity was defined as the amount of enzyme that catalyzed the conversion of 1 μM H_2_O_2_ in one minute.

Total GPX activity was assayed by a method using *tert*-butyl-hydroperoxide and reduced GSH as substrates [58]. The quantity of enzyme that catalyzes the oxidation of one μM of NADPH per minute represents one unit of enzyme activity.

The activity of GR was determined according to the method described by Goldberg and Spooner [59]. One unit of GR activity was calculated as the quantity of enzyme that consumed 1 μM of NADPH in 1 min.

Glucose 6–phosphate dehydrogenase activity was measured according to the method described by Lohr and Waller [60]. The amount of G6PD that reduces one μM of NADP^+^ in one minute defines one unit of enzyme activity.

### 3.5. Oxidative Stress Markers

After the cell lysates were deproteinized with 5% sulfosalicylic acid, GSH analysis was done using the Detect X Glutathione Colorimetric Detection kit (Arbor Assay, Ann Arbor, MI, USA), according to the manufacturer’s instructions. This assay allowed the quantification of both total and oxidized glutathione after blocking the free GSH using 2-vinylpyridine.

Malondialdehyde, an advanced lipid oxidation end product, was assessed by a fluorimetric method, based on fluorescence analysis at *E*x_520nm_/*E*m_549nm_ (Jasco FP750 spectrofluorometer, Jasco Inc., Easton, MD, USA) of the MDA–thiobarbituric acid adduct formed in acidic conditions at 37 °C [61]. A calibration curve with 1,1,3,3-tetramethoxypropane in the range 0.05–5 μM was used to extrapolate the MDA concentration. The results were expressed as nM of MDA/mg protein.

### 3.6. Preparation of Membrane Protein Fraction

Cell membrane enriched fractions were prepared using Membrane II RedyPrep Protein Extraction kit (Bio-Rad, Bio-Rad Laboratories, Hercules, CA, USA) according to manufacturer’s instructions. We replaced 2-D Rehydration/Sample Buffer with membrane rehydration buffer containing Tris-HCl 40 mM, pH 8.5 containing 1 mM EDTA, 4 M urea, 1 mM dithiothreitol, 1:100 Protease Inhibitor Cocktail and 1% (*w*/*v*) Triton X-100.

### 3.7. Western Blotting

For RAGE analysis, the membrane protein fractions were used, while for the other proteins evaluated, the cell lysates were utilised. Briefly, the proteins were separated on 12% polyacrylamide gels in denaturant and reducing conditions and transferred onto polyvinylidene fluoride (PVDF) membranes, which were subsequently blocked using the blocking solution supplied in the Western Breeze Chromogenic Immunodetection kit (Life Technologies, Carlsbad, CA, USA). This kit also provided the washing solution, the alkaline phosphatase-conjugated secondary antibodies, and the chromogenic solution. The monoclonal antibodies used were: anti-RAGE (RD9C 2), anti-HSP 27 (sc-13132), anti-HSP 60 (sc-376261), anti-HSP 70 (sc-24) (Santa Cruz Biotechnology, Santa Cruz, CA, USA) and the monoclonal anti-β-actin (A1978, Sigma-Aldrich, St. Louis, MO, USA). The protein expression levels were quantified with BioCapt 12.6 software (Vilbert Lourmat, Torcy, France), and normalized against β-actin while RAGE expression was normalized against the total proteins blotted [62].

For NF-κB detection, whole-cell protein extracts were resolved on Mini-PROTEAN TGX Stain Free 4%–15% precast gels (Bio-Rad, Bio-Rad Laboratories), transferred onto 2 μm PDVF (V3 Western Workflow, Bio-Rad Laboratories) and digitalised using the ChemiDoc MP System (Bio-Rad Laboratories). Total proteins transferred were quantified using the Image Lab software (version 5.0, Bio-Rad Laboratories) and the membranes were blocked using 5% non-fat dry milk, overnight. NF-κB p65 protein expression was revealed using the polyclonal NF-κB p65 (VPA00015, AbD Serotec, Oxford, UK), HRP conjugated secondary antibody (5184–2504, AbD Serotec, Oxford, UK) and Clarity Western ECL Substrate (Bio-Rad Laboratories). The chemiluminescence was acquired using the ChemiDoc MP System, driven by the Image Lab software. The NF-κB p65 protein expression was quantified using the Image Lab software, and normalized to the total proteins transferred onto the membrane.

### 3.8. SOD Zymography

The activities of Cu/Zn–SOD (33 kDa) and Mn–SOD (88 kDa) were analysed according to a method described by Beauchamp *et al.* [63]. Briefly, cell lysates (40 μg per well) were resolved by PAGE on a 15% PAA gel. The gels were incubated in 2.45 mM nitrotetrazolium blue chloride for 20 min (in the dark), and then incubated in 36 mM potassium phosphate buffer, pH 7.8, with 28 μM riboflavin and 2.8 mM tetramethylethylenediamine for 15 min in the dark. Upon illumination, clear bands indicating superoxide dismutase activity appeared against a dark background. The bands were size-calibrated with a pre-stained molecular mass standard (Life Technology, Carlsbad, CA, USA) and enzymatic activity was quantified using BioCapt 12.6 imaging software (Vilbert Lourmat, Torcy, France).

### 3.9. RNA Extraction and Semi-quantitative Real-Time PCR

After the desired exposure intervals to AGEs–BSA or BSA, HEK 293 cell cultures were harvested and total RNA was extracted using TRIzol reagent (Life Technology) following the Chomezynski method [64]. The RNA concentration and purity ratio were determined by UV absorbance analysis using a BioSpec-nano spectrophotometer (Shimadzu, Kyoto, Japan). RNA quality was assessed using the 2100 Bioanalyzer platform (Agilent, Santa Clara, CA, USA) and the 6000 RNA Nano kit (Agilent). All RNA extracts used had a RIN above 7 [65]. Total RNA extracted was reverstranscribed into cDNA using the Bio-Rad iScript cDNA synthesis kit (Bio-Rad, Hercules, CA, USA). For gene expression analysis, specific primers were designed using the NCBI Database [66] and Primer3 Input software (version 0.4.0) [67,68] (Table 2). As housekeeping gene, *GAPDH* was chosen. Real-time PCR was performed using the iQ SYBR Green Supermix kit (Bio-Rad), with the following temperature cycles: cycle 1 pre-denaturation (10 min, 95 °C), cycle 2 repeated 50 times: denaturation (30 s, 95 °C), annealing (30 s at the annealing temperatures—*T*_a_, indicated in Table 2), and elongation (45 s, 72 °C) steps and cycle 3 that ensures the final extension reaction (10 min, 72 °C). The primers specificity was confirmed by melting curve analysis. The relative gene expression ratio (*R*) was calculated according to the Pfaffl method [69].

**Table 2:** The target genes and the primer sequences used for real-time PCR.

Gene	Accession Number	Oligonucleotide Sequence (5′–3′)	*T*_a_ (°C)	Amplicon Size (bp)
*RAGE*	NM_001136.4	F: TGGATGAAGGATGGTGTG	49	153
		R: GATGATGCTGATGCTGA		
*HSP 27*	NM_001541.3	F: GCCGACATTTGGACACAG	58	184
		R: CTGCTACCTCTGGAGTGG		
*HSP 60*	XM_005246518.1	F: GTGGAAAAAGGAATCATTGACC	58	83
		R: GTAGTTAACAGAGAGGCCACACC		
*HSP 70*	XM_005272813.2	F: AAGGAATTGGAGCAGATGTGTAAC	58	244
		R: CAAGGGATGGTAACTTAG ATTCAGG		
*GAPDH*	NM_002046.4	F: TGGTCTCCTCTGACTTCAAC	58	222
		R: GTGAGGGTCTCTCTCTTCCT		

### 3.10. Statistical Analysis

Data are the mean ± standard deviation (SD) from three independent experiments. An unpaired two-tailed Student’s *t*-test (Quattro Pro X3 software, Version 13.0.0.406, Mountain View, CA, USA) was used to determine the statistical significance, which was marked as follows: *p* < 0.05 was noted with *, *p* < 0.01 was indicated by ** and *p* < 0.001 was labelled with ***.

## 4. Conclusions

HEK 293 cells exposed to AGEs–BSA invoke an interaction between inflammation, oxidative stress and antioxidant systems, which determines the cellular fate. Although AGEs are traditionally known to act via the RAGE receptor, the earliest inflammatory cellular responses we observed did not appear to temporally coincide with RAGE up-regulation. In turn, inflammatory cytokine IL-6 is key to the induction of early inflammatory response in HEK 293 cells. Also, oxidative stress probably contributed to pro-inflammatory signaling activation after the antioxidative systems began to weaken. Notably, HSP 27 inhibition, HSP 70 up-regulation, and the presence of anti-inflammatory cytokines lowered the strength of pro-inflammatory signals, contributing to inflammation regulation in HEK 293 cells exposed to AGEs–BSA for 48 h.
